# Familial Gigantiform Cementoma: Case Report of an Unusual Clinical Manifestation and Possible Mechanism Related To “Calcium Steal Disorder”

**DOI:** 10.1097/MD.0000000000002956

**Published:** 2016-03-07

**Authors:** Chunyue Ma, Hongwei Wang, Guang He, Xingjun Qin

**Affiliations:** From the Department of Oral & Maxillofacial – Head & Neck Oncology, Ninth People's Hospital, Shanghai Jiao Tong University School of Medicine, Shanghai Key Laboratory of Stomatology (CM, HW, XQ), and Bio-X Institutes, Key Laboratory for the Genetics of Developmental and Neuropsychiatric Disorders (Ministry of Education), Shanghai Jiao Tong University (GH), Shanghai, China.

## Abstract

Familial gigantiform cementoma is an exceedingly rare but distinct subtype of cemento-osseous-fibrous lesion. Undocumented radiographic changes and related bone metabolism disorder are herein hypothesized and discussed.

We present an adolescent case with recurrent familial gigantiform cementoma who received surgical intervention in our hospital. Apart from typical multiquadrant and expansile abnormalies involving both jaws, he also suffered from several times of fractures in lower extremity. Furthermore, radiographic examinations of calvaria, pelvis, femoris, tibia, and fibula all revealed radiolucent areas signifying diffuse osteopenic bone losses. Some of his consanguineous relatives bore the same burden of fractures during pubertal period.

Considering these polyostotic conditions, a correlation of congenital bone metabolism disorder in cases with familial gigantiform cementoma, named “calcium steal disorder,” was thus proposed.

Familial gigantiform cementoma is closely associated with “calcium steal disorder.” Whole-body dual-energy absorptiometry should be considered as a routine examination for fracture-related risk prediction.

## INTRODUCTION

Familial gigantiform cementoma (FGC) is a distinct and uncommon fibro-cemento-osseous lesion with unknown etiology. According to the latest World Health Organization (WHO) classification of cemento-osseous dysplasias (CODs), FGC is generally characterized by rapid osseous expansion involving all 4 jaw quadrants with predilection for young patients. Besides, it follows an autosomal dominant inheritance pattern with divergent phenotypic expression.^1^ Although nearly indistinguishable from conventional CODs since the early onset, FGC tends to reveal a locally aggressive feature similar to neoplastic growth, which will ultimately cause severe malocclusion and pronounced facial deformity. As far as our knowledge is concerned, this pathognomonic trait thus necessitates timely and appropriate surgical treatment to prevent these terrible clinical scenarios from spinning out of control.^2^

Genetically predisposing as FGC seems, emphasis of management has largely been confined to mostly afflicted maxillas and mandibles. However, extragnathic presentations of FGC in patients have often been neglected, owing in part to rarity of this disease. Among all the reports available, Rossbach et al^3^ was the first to postulate the correlation of a brittle bone disorder with FGC. Nevertheless, in terms of his description, the progression of disease and related underlying causes has not been clarified. Therefore, we present a case with a large FGC family history so as to sketch a more detailed portrait of such ailment. The constitution of these different family members representing all age spectrums draws us a clearer outline of ‘calcium steal disorder’ for disease progression, which will then be elaborated as well. The ethical approval was granted by the Institutional Clinical Research Supervision Committee of our hospital. Besides, the informed consent was obtained from these patients.

## CASE REPORT

**FIGURE 1:**
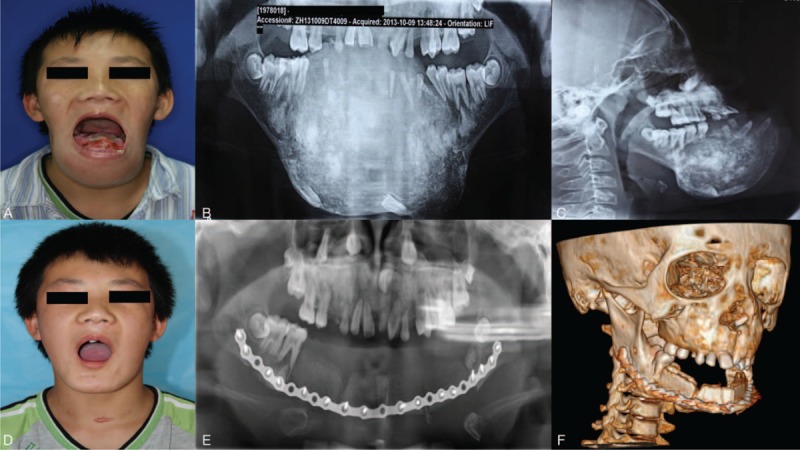
(A) The adolescent patient with a huge mass extending along the mandible body. (B) Preoperative panorex X-ray showed a characteristic radiographic feature of familial gigantiform cementoma (FGC) with well-circumscribed radiopaque areas involving all quadrants of the jaw, with mandible being more severely damaged. (C) Lateral view of FGC. (D) Postoperative view of patient after bilateral mandibulectomy. (E) Postoperative panorex X-ray showed vascularized iliac bone flap for reconstruction. (F) 3-D computed tomography reconstruction of postoperative view.

A 14-year-old male patient was referred to our hospital for recurrence of a huge benign tumor in oral cavity. His chief concern was dysmasesis and dysphagia caused by a protuberant mandible since 10 years old. Asymptomatic as it appeared in early stage, a gradual and incessant enlargement involving the entire corpus of mandible had caused severe facial disfigurement (Figure 1). Surgery with recontouring and shaving intent was initially attempted in a local institution about 7 months ago, but in vain. The growth of the disfigured mandible had not yet been deterred, but instead, accelerated, accompanied by teeth loss and altered dietary patterns. As a direct consequence, physical deterioration was also found by his parents.

**FIGURE 2:**
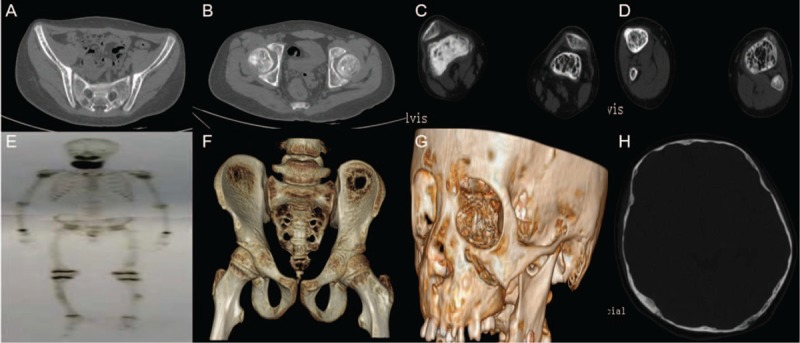
(A & B) Axial computed tomography (CT) scan revealed that coccygeal vertebra, ilium and femur neck were all undergoing osteopenic changes. (C & D) Radiolucent images, with foamy trabeculae and thinner cortex of femur, patella, tibia, and fibula. (E) Technetium scintigraphy showed increased tracer uptaker in the chin area. (F) Two bony defects were identified in reconstructed pelvic CT images. (G & H) CT showed loss of lamina dura, decreased skull bone density, and sporadic bony defects.

His previous surgical history was remarkable and extensive. Before referral, he had experienced 4 times of fractures in bilateral lower extremities within 4 years. All these fractures, which occurred in diaphyseal locations, were categorized to a minor-trauma or spontaneous causes owing to poor evidence of outside forces. The radiographic evaluations of lower limb revealed decreased bone density and thinner- or void-cortex structures around fracture regions. Femur, fibula, tibia, together with patella were affected with osteopenic or absorptive changes (Figure 2). Suspicious of similar osseous problems in other anatomic locations, we then recommended a pelvic computer tomography (CT) scan to the patient. Based on findings garnered from CT, it was noteworthy that 2 big circular deficits could be easily detected on both sides of the iliac bones. Besides, his pelvis was also considered to be susceptible to fractures because both cortical and trabecular bones were undergoing a progeric or osteopenic conversion signifying an unexpected calcium loss. Although panoramic X-ray result exhibited a typical expansile FGC image with predominantly radiopaque signals in all 4 jaw quadrants, it is quite contrary to our thoughts that the other adjacent skull bones, such as temporal and occipital bones, displayed osseous hypoplasia signs (Figure 2). Loss of lamina dura, decreased skull bone density, and sporadic bony defects all served as convincing evidence of the serious osseous fragility. Technetium scintigraphy showed increased tracer uptaker only in the chin area (Figure 2). To further identify the specific reasons for multiple fractures alongside these osseous changes previously described, whole-body dual-energy absorptiometry (DEA) measuring bone mineral density (BMD) was then offered under permission of the patient and his parents. A shape decrease in *Z* score was ascertained by the result, indicative of systemic BMD loss, which is equivalent to 3/4 (72.95%) of his counterparts of the same age.

For sake of differential diagnosis with hyperparathyroidism-jaw tumor (HPT-JT) syndrome,^4^ several laboratory analyses, which included serum parathyroid hormone (PTH), phosphate levels, calcium levels, and alkaline phosphatase (ALP) activity, were undertaken accordingly. All these results came out with no marked aberrance. The feasibility of using fibular flaps was ruled out in view of his unfortunate history of frequent lower-extremity fractures. In concurrence with the boy and his parent's wishes, we decided to perform a bilateral segmental mandibulectomy and to tentatively reconstruct the corresponding defect with vascularized iliac bone flaps. During 11-month follow-up afterwards, the young patient experienced another physical blow of minor-trauma fracture near the femur neck. Since then, supplementation of calcium and vitamin D had been prescribed as a method to ameliorate the general calcium metabolism disorder. For the latest visit to our clinic 2 months ago, the outcome in the neomandible region was desirable, and in parallel, the recent DEA test result took a favorable turn as BMD had increased to 81.26% of his counterparts.

**FIGURE 3:**
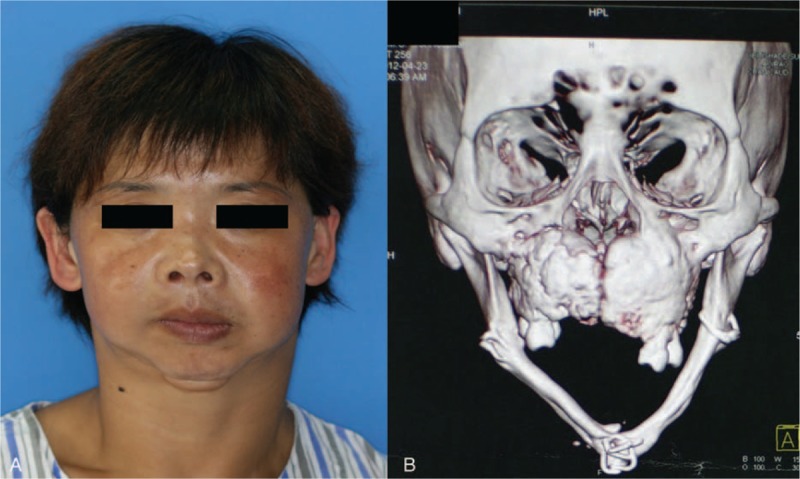
(A) The adolescent patient's aunt who had received surgery (nonvascularized iliac bone reconstruction) 30 years ago now complaint of anterior maxillary mass with chronic infection. (B) Reconstructed computed tomography image of familial gigantiform cementoma appearance.

Misfortunes and troubles never come singly. Closer examination of his family pedigree verified our concern of a long-standing phenomenon of multiple fractures accompanying FGC in jaws. As was revealed in Table 1, a huge number of his relatives had as well experienced multiple fractures during pubertal or prepubertal period. The patient's aunt, who suffered from local infections caused by FGC in the maxilla, also received bilateral subtotal maxillectomy in our hospital (Figure 3). She was yet no exception given her own narratives of femur fracture during adolescence.

**TABLE 1:**
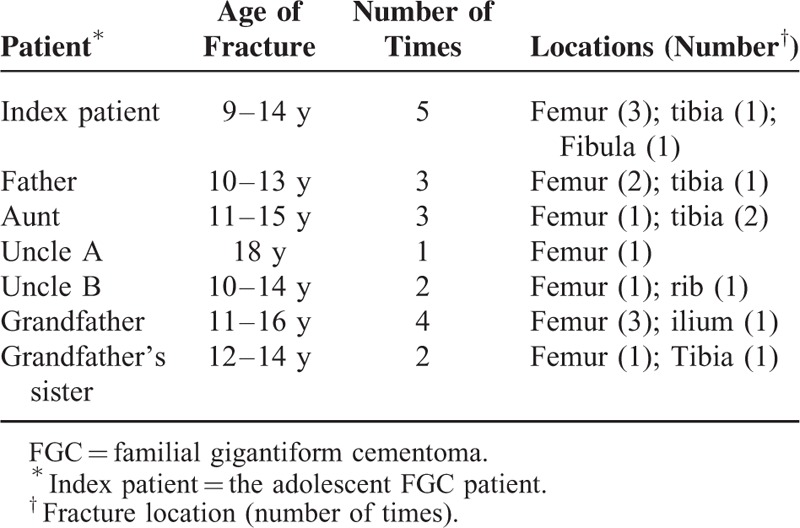
Summary of Extragnathic Fractures of the FGC Pedigree

## DISCUSSION

According to the 2005 World Health Organization classification of osseous dysplasias (OD), FGC is generally regarded as an odontogenic lesion that shares a same periodontal ligament origin with focal, periapical. and florid OD.^5^ Although FGC is histologically consistent with the other 3 ODs, which are characterized by fibroblastic tissue proliferation intermixed with predominantly “cementum-like deposits,” it is yet a quite distinct clinical entity.^6^ The use of diagnostic term “familial” in FGC is warranted for its autosomal dominant transmission feature and a predilection for younger age. In addition, FGC also carries a striking tendency toward more exuberant growth with multiquadrant jaw involvement.^1,7^

The knowledge concerning FGC has been broadened for the recent proposition of the possible correlation with polyostotic diaphyseal fractures.^3^ Despite the original belief that FGC is strictly confined to facial bones, Rossbach et al observed and described 4 individuals of an African-American family with multiple tibial and fibular fractures history. Circumstantial evidence and thorough reasoning were presented in his article for differential diagnosis with other easily mistaken diseases, such as Paget disease, cemento-ossifying fibromas and osteogenesis imperfecta. In 2008, Moshref et al^8^ again reported a FGC case series with frequent fracture history. He surmised that this phenomenon was merely because of genetic heterogeneity and not every case would develop such obvious concurrence of FGC and fractures. Admittedly, not every FGC case will present such evident array of disorganized conditions. We might not hastily come to the conclusion that coexistence of FGC and polyostotic pathologic fractures is mere coincidental clinical manifestation.^8^ However, when we started to combine all the pieces of information at hand, a comprehensive image of FGC progression began to unfold itself. During the rapid growth phase of FGC in mandible and maxilla, the other bones, especially long bones in lower extremities, were simultaneously undergoing a calcium absorption or transportation problem, as was reflected in the osteoporotic radiographic changes in our adolescent patient. The sharp contrast of CT images between densely bony deposits in FGC lesion and the radiolucent low-density images confirmed our hypothesis that both maxilla and mandible of FGC shared unevenly and favorable distribution of calcium deposits in the general calcium metabolism of whole body. Hence, in light of this finding, we came up with the term “calcium steal disorder” for summary. This concept was actually borrowed from “blood steal phenomenon” observed in some arteriovenous malformation cases.^9,10^ As interpreted by Kelly et al^9^ for “blood steal phenomenon,” shunting of high-volume arterial blood through low-resistance arteriovenous fistulae would result in venous hypertension, hypoperfusion of arterial vessels and tissue downstream, and reduced cerebral perfusion. In similar manner, large FGC lesion tends to plunder most calcium deposits and thereby extend itself with osseous growth. However, owing to such diversion of calcium supply, bone fragility was manifested in the other anatomic regions, especially in lower extremities. To keep both practitioners and patients informed of the overall bony changes and corresponding risks of fractures, we contended that DEA should be routinely tested since initial clinic visit. As for prognosis of “calcium steal disorder,” we reckon that: it is quite reversible once under appropriate surgical management for local FGC lesions; although the systemic osseous disorder tends to exacerbate in prepuberty or puberty period, it seems to embark on a gradual process of remission after puberty, as is revealed in our pedigree of patients; calcium plus vitamin D supplementation, which is frequently prescribed and pursued in osteoporosis treatment, may well be applied to FGC patients as part of a comprehensive treatment plan; whether BMD has increased or not in DEA testing may serve as an auxiliary measure of surgical success during follow-up.^11^ So, from our perspective, FGC should rather be considered as a polyostotic bone disease sui generis with calcium metabolism disorder, although still under the heading of fibro-osseous lesions.

Compared with other 3 CODs, FGC takes on a really unique and aggressive form of behavior that is not supposed to be clinically approached in the existing framework of classification. The very fact that most CODs become static or quiescent after skeletal maturation has created a false impression that a more permissive or laissez-faire attitude toward all CODs should be indiscriminately justified for seemingly “self-contained” characteristics.^12^ Some indicated that active treatment is not always necessary, whereas trimming of the affected bone might be necessary from a cosmetic perspective.^13^ Benign as FGC appears from a pathologic perspective, caution and vigilance should be given to treatment since a conservative approach, such as surgical trimming and contouring, will more often than not lead to local recurrence and stimulation of tumor growth, as is presented in our patient. Therefore, we agree with Noffke et al^14^ and Finical^15^ in resorting to a complete resection of FGC with curative purposes whenever feasible. Incomplete excision or shave-off contouring is not advised because it may cause a possible aggravation or reactivation of rapid FGC growth. In such scenarios, extensive resection and free-flap reconstruction are required to achieve a better outcome. Apart from that, it is still debatable about the timing of surgical intervention for FGC patients. A review of latest articles showed that mandible remains to be the most seriously affected organ, in contrast to maxillary lesion.^1–3,8^ Patients with lesions undergoing chronic osteomyelitis or accelerated growth should resort to complete surgery, whereas a close “wait-and-see” approach should be considered firsthand for those with inconspicuous symptoms.

## CONCLUSIONS

To sum up, our unusual radiographic and clinical findings of FGC give rise to a renewed understanding and a broad change to the stereotypic definition. “Calcium steal disorder” should always be kept in mind for such cases. Despite the paucity of information regarding FGC, DEA, as we believe, carries diagnostic and therapeutic implications, along with other radiographic examinations.
